# Exploring background risk factors for fatigue crashes involving truck drivers on regional roadway networks: a case control study in Jiangxi and Shaanxi, China

**DOI:** 10.1186/s40064-016-2261-y

**Published:** 2016-05-10

**Authors:** Changkun Chen, Jun Zhang

**Affiliations:** School of Highway, Chang’an University, Middle Section of South 2 Ring Rd., Xi’an, 710064 China

**Keywords:** Fatigue crash, Truck drivers, Risk factor, Case control study, Pearson Chi-square test, Stepwise logistic regression

## Abstract

**Background:**

Fatigue driving is a leading cause of traffic fatalities and injuries in China, especially among heavy truck drivers. The present study tried to examine which and how factors within the human-vehicle-roadway-environment system contribute to the occurrence of crashes involving fatigued truck drivers.

**Findings:**

To reduce such risk on the road, a total of 9168 crashes which occurred in Jiangxi and Shaanxi between 2003 and 2014 were selected to measure the effects of potential factors on fatigue related truck crashes using a case control study. Pearson Chi-square test was used to determine the relationship between crash risk and independent factors, and a stepwise logistic regression model was developed to determine the significant risk factors. According to the data analysis results, driver’s gender, age, driving experience, and overspeeding behavior, vehicle’s commercial status, overloading conditions and brake performance, road’s type, slippery pavement and existence of sharp curve and long steep grade, and time of day, season, weather and visibility conditions, etc. were identified to be significantly associated with fatigue related truck crashes on Jiangxi and Shaanxi highways. Moreover, it is found that (a) in Jiangxi, an employed truck driver has a higher risk of crash involving multi-vehicles or a passenger car at bridge locations, and (b) in Shaanxi, the adult, tunnel location, summer and winter days prohibit statistically significant association with the occurrence of multi-vehicle and single-vehicle run-off-road/rollover crashes.

**Conclusions:**

Young employed male truck drivers with less experience are at high risk, especially while driving across sharp curves, down long steep grades, over bridge or through tunnels, during the midnight period, on rainy, snowy or foggy days in rural areas. All these help recommend potential policy initiatives as well as effective safety promotion strategies at the public health scale for professional truck drivers.

## Background

Any crash caused by drivers’ fatigue, including falling asleep behind the wheel during driving, is categorized as a fatigue-related crash. During the past two decades, the rapid expansion of economy and urbanization has led to a dramatic increase in truck use in China, and the number of registered trucks has grown from about 20 million in 2005 to more than 32 million in 2013, according to China Statistical Yearbook 2014. Specifically, trucks are often used for commercial purpose, and thus the truck drivers must remain focused behind the wheel for long hours, which can easily cause fatigue (Di Milia 2006; Romo et al. 2014). When driving long distances, especially at night, a truck driver is more likely to experience tiredness, which can cause an involuntary withdrawal of attention from the road ahead, extended reaction time, slower responses to danger, and falling asleep at the wheel. All of these symptoms induce important performance degradation, and thus increase the likelihood of crashes (Chu 2012; Islam et al. 2014). It is reported that trucks account for only 7.8 % of all motor vehicles, but have caused 17.5 % of total road crashes as well as 22 % of total deaths in China (Chen and Xie 2014). Addressing the high risk of road accidents for this huge group, therefore, represents an important public health issue for the population.

Given the human, social, and economic impacts of these crashes worldwide, it is essential to ascertain the crash characteristics and determine the risk factors in order to better understand how and why they happen (Pei and Fu 2014). In previous studies, drivers’ demographic characteristics are a significant contributory factor affecting fatigue-related truck crashes (Di Milia et al. 2011). Among these factors, young male drivers with less driving experience exhibited the highest risk for being involved in fatigue-related crashes (Davenne et al. 2012). This is perhaps because male drivers are often overconfident in their driving skills and therefore, they exhibit dangerous behaviors frequently (Shinara and Comptonb 2004), such as reckless driving, fatigued driving, and drunk driving, etc. In addition, young, inexperienced drivers are also considered high risk, because they often underestimate the potential risks associated with driving situations, and therefore, they cannot tackle driving challenges properly (Friswell and Williamson 2013; Wang et al. 2014). A driver’s occupation is another risk factor that affects the occurrence of fatigue-related crashes (Rodriguez et al. 2003). Truck drivers usually have to face stressful working conditions, with long work hours and tight schedules (Arnold et al. 1997; Cœugnet et al. 2013), such as night time driving, irregular rest times, etc., and even do the non-driving physical work (i.e. loading, unloading, heavy lifting, etc.), too. As a result, they have an insufficient amount of time to rest and recover between shifts, which can easily lead to fatigue (Di Milia 2006; Choi et al. 2014).

Likewise, roadway geometrics and environmental factors significantly affect the likelihood of fatigue crashes (Friswell and Williamson 2013; Crum et al. 2001; Jamroz and Smolarek 2013; Du et al. 2015). Monotonous driving situations, with less demanding tasks, would increase fatigue; thus, drivers can easily get fatigued when driving for a long time on straight, smooth and low-traffic roads (Thiffault and Bergeron 2003). Moreover, each individual has his/her own internal “body clock,” also known as “biological rhythm”, continuous driving when one should be sleeping leads to degraded performance, especially during from 22:00 to 6:00, which indicates that the time of day is an important risk factor (Di Milia and Kecklund 2013). Certainly, drivers’ fatigue patterns should be studied further, due to their significant impact on driving performance. In addition, poor driving conditions, such as hot, rainy, or foggy weather and noise, etc., can also affect the driver’s concentration, and thus are more likely to increase fatigue (Chipman and Jin 2009).

**Fig. 1:**
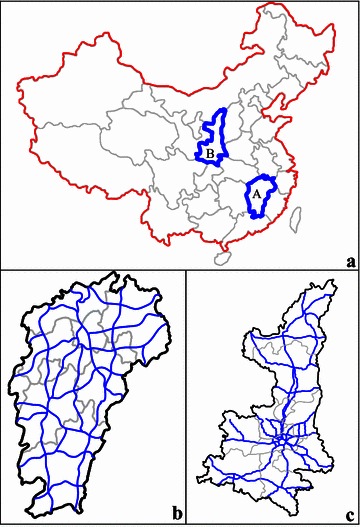
Geographical distribution of expressways in JiangXi (*A*) and Shaanxi (*B*), China. *Note*: Jiangxi, also called “Gan” for short, is located in the southeastern part of Mainland China, on the southern bank of the Yangtze River, covering a total area of 166,900 sq km with a population of about 45.42 million by the end of 2014. Now, it has a total provincial road network of 155,515 km, divided in five administrative levels: 4515 km of expressway (**b**), 1902 km of a first-class highway, 9941 km of a second-class highway, 10,619 km of a third-class highway, and an additional 101,315 km of a fourth-class highway. As one of the cradles of Chinese civilization, Shaanxi is located along the middle reaches of the Yellow River and a gateway to northwest China. It has an area of 205,600 sq km and its total population reaches 37.75 million by end of 2014. Nowadays, the registered motor vehicles in Shaanxi amounted to more than 6.96 million, and the total highway network exceeds 167,000 km, which includes 4512 km of expressway (**c**), 1235 km of first class highway, 9268 km of second class highway, 18,164 km of third class highway, and 120,445 km of fourth class highway

Despite various efforts, however, many problems related to fatigue-related truck crashes remain unsolved today. Few previous studies focus on an impact analysis of the various factors that contribute to fatigue-induced crashes among heavy trucks in China. Using reported and reliable crash data from Jiangxi and Shaanxi over a recent 10-year time frame, therefore, this study aims to determine the specific background risk factors associated with (a) the occurrence of fatigue-related truck crashes and (b) the severity of injuries caused by such crashes, and then to propose the corresponding traffic legislation and technical measures targeting different truck groups to help reduce injuries caused by road crashes. Due to these two provinces’ specific geographical positions (as shown in Fig. 1), crash features, corresponding risk factors, traffic flow characteristics, and driving environments are more representative when compared with data from 29 other provinces, and thus current studies focusing on Jiangxi and Shaanxi’s road safety and corresponding policy recommendations will help develop nationwide traffic policies and legislative measures.

## Methods

This paper utilizes 12 years of crash data between 2003 and 2014 from the “traffic accident database” (TADS), released by the Ministry of Public Security of the People’s Republic of China and maintained by Jiangxi and Shaanxi Transport Policy Bureau. This system provides precise crash messages that include the time, location, driver and vehicle features, major causes, and environment factors of crashes. Figure 1 presents the location of two studied provinces (Jiangxi and Shaanxi) in China.

The original crash data in TADS consists of 71,695 officially reported crashes in Jiangxi and 63,484 crashes in Shaanxi, among which 12,583 records involved at least one truck. Since the primary purpose of this study is to examine fatigue-related crashes with fatal injuries and fatalities among large trucks, then 9168 records (i.e., 5447 cases from Jiangxi and 3721 cases from Shaanxi) were selected for further analysis based on the original judgment on driving fatigue from traffic police officers, accounting for 6.78 % of total observations.

According to the crash data, the injury severity of each individual involved is coded on a four—point ordinal scale: (1) fatal injury, (2) serious injury, (3) moderate injury, and (4) slight injury. Fatal injury refers to crashes that result in immediate death or subsequent death within 30 days of the accident; serious injury refers to crashes that require hospitalization for 2 weeks or more; moderate injury refers to crashes that require hospitalization from 1 day to 2 weeks; and slight injury refers to crashes that require hospitalization for 24-h or less, or no hospitalization at all.

A case control study was conducted using the selected crash sample to examine the potential factors that may contribute to fatigue-related truck crashes, in which ‘case’ refers to crashes caused by fatigued driving, and ‘control’ refers to crashes caused by other reasons. Contingency table test methods are used to statistically examine the distribution of each explanatory variable and model the relationship between crash characteristics and contributory factors through Pearson Chi-square (χ^2^) test. In addition, we conducted a stepwise logistic regression to measure the effects of different factors that contribute to the likelihood of crash occurrences, to identify the significant factors and examine the effect magnitude of adjusted odds ratios for each risk factor, while controlling for other factors. The adjusted odds ratios and the corresponding 95 % confidence intervals are determined by the stepwise logistic regression model, in which all risk factors are initially entered and the insignificant factors were subsequently removed by the stepwise procedure with probability of 5 %.

Table 1 summarizes the distribution of crash sample characteristics examined in this study, including driver factors, vehicle factors, road factors, environmental factors, and crash characteristics.

**Table 1:** Distribution of crash sample characteristics

Risk contributes	Jiangxi (*N* = 5447)		Shaanxi (*N* = 3721)	
	Frequency	%	Frequency	%
*Driver factors*				
Gender				
Male	5014	92.05	3498	94.01
Female	433	7.95	223	5.99
*Marital status*				
Single/divorced/widow	389	7.14	182	4.89
Living with a partner	5058	92.86	3539	95.11
Age^a^				
Young	1031	18.93	556	14.94
Adult	3504	64.33	2674	71.86
Old	912	16.74	491	13.20
Driving experience in years^b^				
Novice	1594	29.26	923	24.81
Practician	2432	44.65	1928	51.81
Proficient	1053	19.33	653	17.55
Expert	368	6.76	217	5.83
Driver’s occupation				
Employed workers	4979	91.41	3291	88.44
Self-employed	367	6.74	364	9.78
Military and policy	30	0.55	14	0.38
Others	71	1.30	52	1.40
*Vehicle factors*				
Vehicle type				
Goods vehicles	5146	94.47	3511	94.36
Other vehicle types	301	5.53	210	5.64
Commercial transport				
Yes	5154	94.62	3495	93.93
No	293	5.38	226	6.07
Vehicle insurance				
Yes	5058	92.86	3343	89.84
No	389	7.14	378	10.16
Vehicle safety state				
Fit	4791	87.96	3318	89.17
Unfit	656	12.04	403	10.83
Vehicle brake performance				
Good	4285	78.67	3009	80.87
Poor	1162	21.33	712	19.13
*Roadway factors*				
Road type^c^				
Expressway	2262	41.53	1384	37.19
The first class highway	1567	28.77	984	26.44
The second class highway	1053	19.33	813	21.85
The third class highway	226	4.15	188	5.05
The fourth class highway	47	0.86	96	2.58
Rural road	28	0.51	41	1.10
Urban road^d^	264	4.85	215	5.78
Highway specific locations^e^				
Sharp curve	1087	19.96	985	26.47
Long steep grade	2361	43.34	1827	49.10
Interchange	49	0.90	28	0.75
Highway ramp	112	2.06	131	3.52
Weaving section	317	5.82	247	6.64
Tunnel	429	7.88	825	22.17
Bridge	833	15.29	351	9.43
Toll station	33	0.61	22	0.59
Urban road specific locations^f^				
Intersection^g^	104	39.39	123	57.21
Elevated road	13	4.92	19	8.84
Ramp	21	7.95	15	6.98
Under-crossing tunnel	62	23.48	44	20.47
Others	69	26.14	35	16.28
Pavement condition				
Dry	3631	66.66	2548	68.48
Wet	1816	33.34	1173	31.52
*Environment factors*				
Time of crash				
0 am–6 am	1907	35.01	1211	32.55
6 am–8 pm	2661	48.85	1824	49.02
8 pm–12 pm	879	16.14	686	18.44
Day of the week^h^				
Weekdays	4339	79.66	3076	82.67
Weekends	1108	20.34	645	17.33
Seasons^i^				
Spring	936	17.18	347	9.33
Summer	1869	34.31	1422	38.22
Autumn	577	10.59	326	8.76
Winter	2065	37.91	1626	43.70
Public holiday^j^				
Yes	189	3.47	79	2.12
No	5258	96.53	3642	97.88
Weathercondition				
Fine	3725	68.39	2859	76.83
Adverse	1722	31.61	862	23.17
Visibility condition				
Good	3950	72.52	3108	83.53
Bad	1497	27.48	613	16.47
*Traffic violations* ^k^				
Alcohol-impaired driving				
Drunk	281	5.16	167	4.49
Not drunk	5166	94.84	3554	95.51
Disregarding the speed limits				
Speeding	3871	71.07	2976	79.98
Not speeding	1576	28.93	745	20.02
Overloading behavior				
Overloading	3672	67.41	2405	64.63
Not overloading	1775	32.59	1316	35.37
Risky following				
Keep unsafe distance	1716	31.50	1359	36.52
Keep safe distance	3731	68.50	2362	63.48
Failure to use seat belt				
Yes	1033	18.96	862	23.17
No	4414	81.04	2859	76.83
Cell phone use				
Use	582	10.68	589	15.83
Not use	4865	89.32	3132	84.17
Illegal meeting				
Performed	649	11.91	337	9.06
Not performed	4798	88.09	3384	90.94
*Crash characteristics*				
Injury outcome				
Slight injury	849	15.59	533	14.32
Moderate injury	1327	24.36	1188	31.93
Serious injury	1775	32.59	1083	29.11
Fatal injury	1496	27.46	917	24.64
Type of collision				
Head-on collision	1181	21.68	932	25.05
Sideswipes	1427	26.20	1130	30.37
Rear-end collision	1326	24.34	803	21.58
Rollover	408	7.49	171	4.60
Runoff	944	17.33	606	16.29
Others^l^	161	2.96	79	2.12
Collision partner				
A single truck	846	15.53	696	18.70
A truck and a passenger vehicle^m^	1378	25.30	451	12.12
Two trucks	307	5.64	282	7.58
Multiple vehicles^n^	2735	50.21	2117	56.89
Truck(s) and vulnerable road user(s)^o^	181	3.32	175	4.70

^a^Divided into three groups: young (≤30 years old), adult (30–55 years old), and old (≥55 years old)

^b^Divided into four groups: novice (≤3 years), practician (3–5 years), proficient (6–15 years), and expert (≥15 years)

^c^According to the Technical Standards for Highway (JTG B01 2003), released by the Ministry of Transport of the People’s Republic of China in 2003

^d^Including urban expressways, arterials, sub-arterials and minor streets and roads

^e^Frequent occurrence locations of crashes of highways are also known as “black spot”. Here a crash may be attributed to two or more contributing factors. For example, a truck runs off the road at a sharp curve while driving along a long steep grade

^f^Crashes associated with one roadway factor/total crashes occurring on urban roads

^g^Including road level crossing (with and without signal controlled), under-crossing, and interchange

^h^Weekends = 17:00 Friday to 24:00 Sunday; weekdays = 0:00 Monday to 16:59 Friday

^i^Spring = March to May; summer = June to August; autumn = September to November; winter = December to February

^j^Including New Year, Chinese New Year, Qing Ming Festival, International Labor Day, Dragon Boat Festival, Mid-Autumn Festival, and National Day

^k^Only typical risky driving behaviors were included in this study, based on the original TADS records

^l^Including those involving vulnerable road users, etc

^m^Passenger vehicles refer to passenger cars and coaches; Here coach involved crashes account for a very small percentage in crash sample

^n^Referring to three vehicles or more with at least one truck

^o^Including pedestrians, bicyclists (considering those on electric bikes or low speed vehicles such as scooters, mopeds and agricultural tricycles et al.), motorcyclists or pedal cyclists

## Empirical results and findings

### Demographics characteristics of fatigue related truck crashes

As listed in Table 1, the characteristics of fatigue related truck crashes in Jiangxi and Shaanxi are generally similar.

Both truck drivers’ age and gender are important factors that are associated with their crashes. The sample has an extremely high proportion of males, accounting for 92.05 and 94.01 % of the total number of crashes for Jiangxi and Shaanxi, respectively, and about two-thirds of truck drivers are 30–55 years old, making up the largest population group of drivers. In view of driving experience, it can be inferred that the more driving years that truck drivers have, the less likely they are to be involved in fatigue crashes (crash proportion: ‘below 3 years’ = 29.26 and 24.81 %; ‘between 3 and 5 years’ = 44.65 and 51.81 %; ‘between 6 and 15 years’ = 19.33 and 17.55 %; and ‘above 15 years’ = 6.76 and 5.83 %, for Jiangxi and Shaanxi, respectively). Additionally, employed workers are found to have an increased risk of truck crashes while driving when fatigued, and we estimate this group to make up 91.41 and 88.44 % of the total truck crashes in Jiangxi and Shaanxi, respectively.

For vehicle factors, vehicle type, commercial transport status, insurance, overloading conditions, safety status, and brake performance have a positive correlation with fatigued-related truck crashes. Specifically, goods vehicles (94.47 and 94.36 %), commercial transport status (94.62 and 93.93 %), unfit safety status (12.04 and 10.83 %), and poor brake performance (21.33 and 19.13 %) are important factors that significantly affect the probability of fatigue-related truck crashes.

Among road factors, both the type of road and design geometrics show a strong association with fatigue related truck crashes. As shown in Table 1, a large proportion of such crashes occur on expressways (41.53 and 37.19 %), the first class highways (28.77 and 26.44 %) and the second class highways (19.33 and 21.85 %). In particular, they are more likely to occur on curves, grades, bridges, in tunnels or at urban intersections (highway crash proportion: ‘sharp curve’ = 19.96 and 26.47 %; ‘long steep grade’ = 43.34 and 49.10 %; ‘bridge’ = 15.29 and 9.43 %; and ‘tunnel’ = 7.88 and 22.17 %. urban road crash proportion: ‘intersection’ = 39.39 and 57.21 %; and ‘under-crossing tunnel’ = 23.48 and 20.47 %). Moreover, pavement conditions are significantly associated with the proportion of such crashes (crash proportion: ‘dry’ = 66.66 and 68.48 %; and ‘wet’ = 33.34 and 31.52 %).

The results indicate that the time of day is one of the most significant contributing factors in fatigue-related truck crashes. Such crashes are more likely to take place during the evening or at dawn (crash proportion: ‘0:00–6:00’ = 35.01 and 32.55 %; and ‘20:00–24:00’ = 16.14 and 18.44 %), presumably due to the higher level of sleepiness and fatigue associated with human circadian rhythm during these periods. Additionally, a majority of the surveyed crashes took place on weekdays (79.66 and 82.67 %), but not on public holidays (96.53 and 97.88 %). In particular, truck crashes caused by driving fatigue occurred more frequently under adverse weather conditions (31.61 and 23.17 %), such as wet pavements (33.34 and 31.52 %) and decreased visibility (27.48 and 16.47 %), due to the increased brake distance on slippery pavements. Furthermore, winter and summer demonstrate the highest prevalence of fatigue related truck crashes, and each of these two seasons account for more than 30 % of the total crashes for each province.

As expected, a large proportion of sampled crashes in this study are associated with driver’s traffic violations or risky driving behaviours, such as ‘over-speeding’ (71.07 and 79.98 %), ‘over-loading’ (67.41 and 64.63 %), ‘risky following’ (31.50 and 36.52 %), and ‘failure to use seat belt’ (18.96 and 23.17 %). In addition, some other behaviours are also observed in the fatigue related truck crashes (crash proportion: “drunk driving” = 5.16 and 4.49 %; ‘cell phone use’ = 10.68 and 15.83 %; and ‘illegal meeting’ = 11.91 and 9.06 %). All these require specific treatment.

In view of crash forms, the total proportion of sideswipes, rear-end collisions, and head-on collisions account for approximately 70 % or more (crash proportion: ‘sideswipes’ = 26.20 and 30.37 %; ‘rear end’ = 24.34 and 21.58 %; and ‘head-on’ = 21.68 and 25.05 %) of the outcomes reported in the sample. Notably, fatal crashes in Shaanxi account for only 24.64 % of all of fatigue-related truck crash records, where serious crashes account for 29.11 %, moderate crashes account for 31.93 %, and slight crashes account for 14.32 %, over the surveyed 12 years. However, the conditions become more serious when considering crash injuries and severities in Jiangxi, where the proportion of fatal and serious crashes increased to 27.46 and 32.59 %, respectively; but the proportion of moderate injuries decreased to 24.36 %, while slight crashes remained at 15.59 %, during the same period. Table 1 also shows that more than a half of all fatigue related truck crashes is associated with multiple vehicles (50.21 and 56.89 %), and over one-third (25.30 %) of truck crash records in Jiangxi involves a passenger vehicle, but that in Shaanxi decreases to 12.12 %. Additionally, those causing injuries or fatalities to vulnerable road user account for 3.32 and 4.70 % in Jiangxi and Shaanxi, respectively.

### Risk factors for fatigue related truck crashes in Jiangxi

Table 2 presents the Chi-square test of independence and the results of the association examination between crash occurrences and risk factors, which indicate that drivers’ gender, age, and driving experience, disregarding speed limits and risky following behaviors, vehicle’s type, commercial operations, insurance status, overloading conditions, and brake performance, road’s type, specific locations and pavement condition, time of day, seasons, weather condition, and visibility condition are potential factors that significantly affect the fatigue-related truck crashes in Jiangxi, China.

**Table 2:** Chi-square test of variable independence

Risk contributes	Jiangxi			Shaanxi		
	χ^2^	*p* value	Degree of freedom	χ^2^	p value	Degree of freedom
*Driver factors*						
Gender	21.335**	<0.001	1	17.463**	<0.001	1
Marital status	46.412	0.062	3	31.231	0.157	3
Age	13.167**	<0.001	2	21.117**	<0.001	2
Driving experience	6.493**	0.003	3	7.062**	<0.001	3
Occupation	5.615	0.186	6	8.133	0.105	6
*Vehicle factors*						
Vehicle type	96.434**	<0.001	1	104.212**	<0.001	1
Commercial transport	51.601*	0.035	1	77.465*	0.008	1
Vehicle insurance	67.293*	0.044	1	44.047	0.092	1
Vehicle safety status	37.307	0.061	1	19.691	0.106	1
Vehicle brake performance	46.269**	<0.001	1	8.673**	<0.001	1
*Roadway factors*						
Road type	117.355*	0.027	6	129.330*	0.016	6
Highway specific locations	56.493**	<0.001	7	42.092**	<0.001	7
Urban road specific locations	9.038	0.103	4	11.767	0.088	4
Pavement condition	11.764**	<0.001	1	2.439**	<0.001	1
*Environment factors*						
Time of crash	23.697**	<0.001	2	84.465*	0.031	2
Day of the week	4.611	0.084	1	6.731	0.089	1
Seasons	7.498*	0.037	3	14.222*	0.011	3
Public holiday	2.176	0.549	1	7.769	0.115	1
Weathercondition	8.335**	<0.001	1	1.005**	<0.001	1
Visibility condition	41.987**	<0.001	1	17.343**	<0.001	1
*Traffic violations*						
Alcohol-impaired driving	33.764	0.298	1	23.646	0.089	1
Disregarding speed limits	11.028**	<0.001	1	17.988**	0.003	1
Overloading	5.169**	<0.001	1	3.497**	<0.001	1
Risky following	22.633*	0.027	1	15.238	0.061	1
Failure to use seat belt	6.961	0.055	1	8.441	0.107	1
Cell phone use	46.376	0.086	1	98.493	0.064	1
Illegal meeting	5.108	0.161	1	4.767	0.055	1
*Crash characteristics*						
Injury outcome	35.469**	<0.001	3	26.894**	<0.001	3
Type of collision	71.336*	0.033	5	89.471*	0.022	5
Collision partner	23.089**	<0.001	4	16.746**	0.004	4

* Significant at 5 % level; ** Significant at 0.5 % level

Table 3 presents the results of estimating the impact of risk factors on fatigue-induced truck crashes by a logistic regression model, in which human, vehicle, road and environment factors all significantly contribute to the occurrence of fatigue related truck crashes in Jiangxi, China. In terms of crash severity and type, such crashes are also identified to have obvious characteristics, as shown in Table 3.

**Table 3:** Adjusted odds ratios (95 % confidence intervals) in stepwise logistic regression analysis

Risk contributes	Jiangxi			Shaanxi		
	SE	Odds ratio	95 % CI	SE	Odds ratio	95 % CI
*Driver factors*						
Gender (base: female)						
Male	0.271	1.846	(1.172, 2.864)	0.145	0.621	(0.376, 1.083)
Age (base: old)						
Young	0.415	0.663	(0.507, 0.851)	0.766	3.867	(2.437, 4.661)
Adult	–	–	–	0.334	0.681	(0.313, 0.946)
Driving experience in years (base: expert)						
Novice	0.692	4.787	(2.986, 7.129)	0.249	1.465	(0.745, 1.420)
Driver’s occupation (base: others)						
Employed workers	0.253	0.565	(0.310, 0.675)	–	–	–
*Vehicle factors*						
Commercial transport (base: no)						
Yes	1.237	1.688	(1.233, 2.825)	0.655	1.328	(0.891, 2.262)
Vehicle brake performance (base: good)						
Poor	1.454	1.817	(1.257, 2.094)	0.826	1.174	(0.907, 1.771)
*Roadway factors*						
Road type (base: rural road)						
Expressway	1.976	4.248	(3.331, 4.795)	1.652	2.468	(1.762, 3.455)
Highway specific locations (base: others)						
Sharp curve	0.343	0.785	(0.512, 1.317)	0.376	0.981	(0.637, 1.785)
Long steep grade	1.364	2.176	(1.108, 2.766)	0.895	1.364	(0.825, 2.007)
Tunnel	–	–	–	1.498	2.176	(1.383, 3.699)
Bridge	0.637	1.419	(1.124, 3.006)	–	–	–
Pavement condition (base: dry)						
Wet	1.626	1.764	(1.433, 1.859)	5.785	8.463	(4.798, 17.285)
*Environment factors*						
Time of crash (base: 6 am to 8 pm)						
0 am–6 am	0.729	0.907	(0.561, 0.976)	0.770	1.418	(1.117, 1.990)
Seasons (base: autumn)						
Summer	–	–	–	0.664	0.847	(0.543, 1.087)
Winter	0.881	1.465	(1.043, 2.557)	1.083	0.764	(0.626, 1.655)
Weather condition (base: fine)						
Adverse	0.260	0.518	(0.284, 0.809)	0.785	0.988	(0.438, 1.097)
Visibility condition (base: good)						
Poor	1.429	1.376	(0.877, 1.740)	1.178	1.496	(1.133, 2.466)
*Traffic violations*						
Disregarding the speed limits (base: not speeding)						
Speeding	1.465	2.349	(1.875, 3.941)	1.625	2.137	(1.339, 4.081)
Overloading condition (base: not overloading)						
Overloading	1.681	0.848	(0.464, 1.223)	1.464	1.209	(1.177, 1.699)
*Crash characteristics*						
Injury outcome (base: slight injury)						
Moderate injury	0.313	0.686	(0.416, 0.988)	0.117	0.464	(0.374, 0.628)
Serious injury	0.744	1.337	(0.754, 1.767)	0.086	0.752	(0.691, 1.476)
Fatal injury	1.163	1.559	(1.145, 2.386)	0.439	1.767	(1.422, 2.897)
Type of collision (base: others)						
Head-on collision	0.176	1.394	(0.933, 2.498)	0.460	1.128	(0.866, 2.743)
Sideswipes	0.698	1.765	(1.174, 3.992)	0.533	1.665	(1.439, 3.078)
Rear-end collision	0.375	1.567	(1.044, 2.363)	1.176	2.061	(1.414, 3.665)
Runoff	–	–	–	0.421	0.764	(0.297, 1.170)
Collision partner (base: two trucks)						
A single truck	0.706	0.911	(0.665, 1.127)	1.142	1.869	(1.394, 2.527)
A truck and a passenger vehicle	1.163	1.455	(0.896, 1.772)	1.068	1.241	(1.076, 1.718)
Multiple vehicles	1.467	1.893	(1.226, 2.674)	1.383	2.057	(0.896, 2.963)
Truck(s) and vulnerable road user(s)	0.658	0.896	(0.623, 0.972)	0.764	0.883	(0.653, 1.450)
Model estimation measures						
Percentage	84.36			82.55		
ROC	0.877			0.863		
*N*	5447			3721		

Specifically, young (‘≤30’: OR 0.663, 95 % CI 0.507–0.851) less experienced (‘novice’: OR 4.787, 95 % CI 2.986–7.129) male (OR 1.846, 95 % CI 1.172–2.864) truck drivers are more likely to be involved in fatigue crashes. Among all occupations, employed (OR 0.565, 95 % CI 0.310–0.675) truck drivers exhibit higher probabilities of involvement in fatigue crashes.

Among vehicle factors, commercial transport status (OR 1.688, 95 % CI 1.233–2.825) and poor brake performance (OR 1.817, 95 % CI 1.257–2.094) are significantly associated with fatigue-related truck crashes in Jiangxi. As seen in Table 3, slippery roads (OR 1.764, 95 % CI 1.433–1.859) increase the likelihood of crashes. There is a much higher risk for fatigue-induced heavy truck crashes on sharp curves (OR 0.785, 95 % CI 0.512–1.317), long steep grades (OR 2.176, 95 % CI 1.108–2.766), and bridges (OR 1.419, 95 % CI 1.124–3.006) rather than on the other locations. In addition, fatigue-related truck crashes are more prone to occur on expressways (OR 4.248, 95 % CI 3.331–4.795).

The modeling results also show that among driver’s violation behaviors, over-speeding (OR 2.349, 95 % CI 1.875–3.941) and over-loading (OR 0.848, 95 % CI 0.464–1.223) significantly increase the risk of being involved in truck crashes. Considering the environmental factors, fatigue-related truck crashes are more likely to happen between 0 am and 6 am (OR 0.907, 95 % CI 0.561–0.976), under poor visibility conditions (OR 1.376, 95 % CI 0.877–1.740), during adverse weather periods (OR 0.518, 95 % CI 0.284–0.809), and on winter days (OR 1.465, 95 % CI 1.043–2.557) in Jiangxi, China.

In view of the crash types and severities, just as Table 3 shows, fatal and serious crashes are more likely to take place than visible crashes (‘fatal crash’: OR 1.559, 95 % CI 1.145–2.386; ‘serious crash’: OR 1.337, 95 % CI 0.754–1.767; ‘moderate crash’: OR 0.686, 95 % CI 0.416–0.988), if they result from driving fatigue. Sideswipes, rear-end and head-on collisions are three more common crash types among all the crashes observations (‘sideswipes’: OR 1.765, 95 % CI 1.174–3.992; ‘rear-end collision’: OR 1.567, 95 % CI 1.044–2.363; ‘head-on collision’: OR 1.394, 95 % CI 0.933–2.498). Additionally, large trucks are more likely to be involved in fatal multiple-vehicle crashes (OR 1.893, 95 % CI 1.226–2.647) under the influence of fatigue, followed by the truck-passenger vehicle crashes (OR 1.455, 95 % CI 0.896–1.772) and single-truck crashes (OR 0.911, 95 % CI 0.665–1.127).

### Risk factors for fatigue related truck crashes in Shaanxi

Upon analyzing the effects of significant risk factors in fatigue-related truck crashes in Jiangxi, a total of 3721 crash samples in Shaanxi were subsequently examined. From Table 2, 15 factors demonstrate a significant association with fatigue-related truck crashes in Shaanxi, including drivers’ gender, age, driving experience and overspeeding behaviour, vehicle’s type, commercial operation, overloading conditions and brake performance, road’s type, specific locations, and pavement condition, time of day, season, weather condition, and visibility condition.

According to the stepwise logistic regression analysis in Table 3, the results exhibit a higher likelihood to be associated with male (OR 0.621, 95 % CI 0.376–1.083), who are less than 55-years-old (‘≤30 years’: OR 3.867, 95 % CI 2.437–4.661; ‘30–55 years’: OR 0.681, 95 % CI 0.313–0.946), novices (OR 1.465, 95 % CI 0.745–1.420). Table 3 also indicates that truck’s commercial transport status (OR 1.328, 95 % CI 0.891–2.262) and poor brake performance (OR 1.174, 95 % CI 0.907–1.771) present higher risk of being involved in fatigue crashes. Among road factors, sharp curves (OR 0.981, 95 % CI 0.637–1.785), long steep grades (OR 1.364, 95 % CI 0.825–2.007), and tunnels (OR 2.176, 95 % CI 1.383–3.699) have a higher likelihood of occurrence of truck crashes.

Table 3 also indicates that driving during 0:00–06:00 (OR 1.418, 95 % CI 1.117–1.990), on summer and winter days (‘summer’: OR 0.847, 95 % CI 0.543–1.087; ‘winter’: OR 0.764, 95 % CI 0.626–1.655) has an increasing probability of truck crashes due to driving fatigue. Moreover, both poor visibility conditions (OR 1.496, 95 % CI 1.133–2.466) and adverse weather (OR 0.988, 95 % CI 0.438–1.097) exhibit significant influence on truck crash occurrence and frequency, according to 3721 audited crash samples from Shaanxi, China. Specifically, such crashes are more likely to happen on expressways (OR 2.468, 95 % CI 1.762–3.455) under slippery pavement conditions (OR 8.463, 95 % CI 4.798–17.285). Similar findings are also found in Jiangxi, China.

Traffic violations are also proved to have a greater probability of resulting in fatal truck crashes. In examining the crash samples of Shaanxi, truck drivers who frequently commit over-speeding (OR 2.137, 95 % CI 1.339–4.081) and over-loading (OR 1.209, 95 % CI 1.177–1.699) behaviors are more likely to be involved in fatigue crashes.

In terms of crash characteristics, fatal (OR 1.767, 95 % CI 1.422–2.897) and serious (OR 0.752, 95 % CI 0.691–1.476) injuries are negatively associated with fatigue-induced truck crashes. Among these crashes, rear-end collisions (OR 2.061, 95 % CI 1.414–3.665), sideswipes (OR 1.665, 95 % CI 1.439–3.078), and head-on collisions (OR 1.128, 95 % CI 0.866–2.743) rank as the top three most common forms of collision leading to injuries and fatalities. Specially, run off the road crashes (OR 0.764, 95 % CI 0.297–1.170) are also found to happen more frequently due to truck driver’s driving fatigue in Shaanxi expressways, especially within the Qinba areas. Moreover, such crashes are most likely to occur involving multiple vehicles (OR 2.057, 95 % CI 0.896–2.963), which keeps consistent with the previous findings in Jiangxi, but those involving single trucks also often happen in run-off-road and rollover crashes in Shaanxi (OR 2.057, 95 % CI .896–2.963).

## Conclusions and discussions

Consistent with present findings in the literature, our results also reveal that the commercial male truck drivers with less experience exhibit a propensity for fatigue crashes, because they are doing a high-risk job and have to drive more often and longer on the road, under time constraints, as well as are more frequent to engage in risky driving behaviours, such as over-speeding and over-loading. All these in turn increase the likelihood of being in a fatigue crash. Specially, employed truck drivers exhibit higher probabilities of involvement in fatigue crashes in Jiangxi, China, because a large percentage of traffic are pass through vehicles, especially the bidirectional traffic between Guangdong Province in South China and North China as well as those between the developed east coast provinces and the remote western areas in China. In Shaanxi, however, such effects are not observed.

**Fig. 2:**
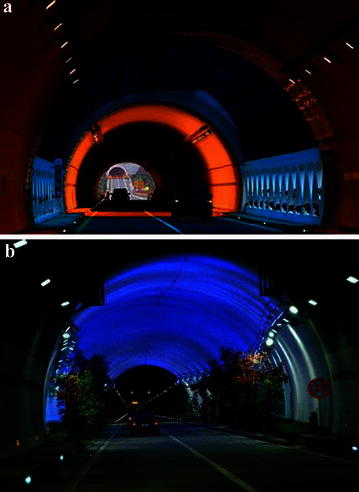
Specific environment design in Zhongnanshan Tunnel of G65 Baotou–Maoming Expressway, China. **a** Connection section of short tunnel; **b** lighting section inside long tunnel

Overall, Jiangxi and the majority of Shaanxi are mountainous regions, and truck drivers in these areas are more prone to fatigue-related truck crashes, because of poor roadway geometric design below existing guidelines, such as sharp horizontal curves, steep grades, and absence of tunnels and bridges, etc. Driving over these road segments, truck drivers have to tackle much more frequent, complex reactions and bear high workloads, which exhaust them more quickly. In the long run, such drivers will get fatigued and insensitive to emergency conditions. If they stay alert by keeping themselves in a proper driving posture (i.e. compliance with load and speed restriction and rules regarding continuous driving, breaks and total daily driving, et al.), however, it is generally possible for commercial truck drivers to have good safety records. On the other hand, we did find that fatigue-related crashes among truck drivers sometimes take place on unchallenging, straight road sections, where drivers can acclimatize to the monotonous environment, gradually fall asleep or get distracted and then lose the necessary arousal and vigilance to potential driving risk, which has important implications for roadway design and alignment. Accordingly, it is worthwhile to set multicolour lighting zones and other types of man-made landscape within long tunnel to change the monotonous environment and relieve fatigue (Wang et al. 2016), as performed in the 18.04 km long Zhongnanshan Tunnel in Shaanxi province, China, a section of G65 Baotou–Maoming Expressway (see Fig. 2).

Truck drivers in Shaanxi are found to have a higher probability of being involved in fatigue-related crashes on tunnel sections, especially on the freeways through the major mountains of Qinling, such as G5 (Beijing–Kunming Expressway). In Jiangxi, however, bridge locations seem to be more dangerous. Among environmental factors, time of day was a negative factor that could increase the risk for fatigue crashes among truck drivers. In particular, overnight and during early morning hours (12 midnight to 6 am) are the periods of highest risk, mainly because this is the time that drivers should be in the bed, rather than in the car. In addition, night-time driving may be associated with potential alcohol involvement or music listening, which can impair driver’s performance or distract his/her attention easily (Arnedt et al. 2001; Vanlaar et al. 2008; Brodsky and Slor 2013). On the other hand, bad weather and poor visibility are significantly associated with fatigue-induced crashes involving trucks, largely because trucks need longer distances to brake safely on slippery roads, thus truck drivers experience the highest risk of crashes on slippery roads; however, both drivers and passengers have a lower injury propensity, presumably because vehicles proceed at slower speeds and drivers are more cautious (Russo et al. 2014).

It was found that head-on, rear-end, and angle collisions are associated with more severe injuries among all occupants, while side-swipe collisions demonstrate a lower severity level for injuries. Multiple-vehicle crashes comprise more than half of all crashes involving at least one truck in rural areas during the time period studied. Under the influence of fatigue, a heavy truck is much more likely to crash with a passenger car in Jiangxi, but in Shaanxi it has the higher risk of being involved in a single-vehicle run-off-road or rollover crashes. Furthermore, those involving vulnerable road users are less likely to occur, which indicates that truck crash associated with fatigue is not a major problem in urban areas.

As expected, a driver should be allowed to drive only as long as his/her ability to drive is not impaired (Zaranka et al. 2014; Wang et al. 2015). Therefore, the government and responsible departments should launch corresponding law enforcement programs to regulate driver’s max weekly and fortnightly driving hours, maximum continuous driving time in 24 h, and standard minimum rest hours after maximum continuous driving, to reduce fatigue-related crashes. Thus, state-of-the-art technology should be developed to evaluate the drivers’ driving performance, provide the warning or alerting messages and assess crash risk, if necessary (Jung and Shin 2014). Specially, drivers are strongly recommended to own vehicles with automatic in-vehicle safety equipment, to alarm drivers of their fatigue or physical impairment, etc.

On the other hand, an education program for safe driving can be developed for not only truck drivers, but also employers. Employers should be forced to extend delivery times and alleviate the burden of truck drivers, and drivers should be provided with intensive awareness training by local governments or employers about how to alert the danger of driving fatigue as well as how to deal with it. Governing agencies could provide local media advertising, cell phone information, brochures or schooling, particularly for long-distance truck drivers, to enhance drivers’ knowledge, skills and ability to drive safely. Specifically, it is strongly suggested that improved services at rest stations and larger parking facilities should be offered to make breaks and rest hours more attractive for drivers.

Overall, our findings provide a thorough understanding of the various contributing factors that have a negative association with fatigue-related truck crashes in Jiangxi and Shaanxi, China, and thus can help improve overall truck safety on road. However, the study has some obvious methodological limitations in nature. First, the crash sample was only selected from Jiangxi and Shaanxi, and may be not representative of the overall situation of traffic safety in China. Second, the original data may potentially contain inaccuracies, due to unreported crashes and injuries, indecipherable hand writing, missing or incomplete messages, etc. Third, there is neither “breathalyzer” nor other direct and reliable tool that an investigating police officer can use to determine whether some level of fatigue was a major or contributory factor in a crash, and thus police findings during the original accident report may underestimate the level of fatigue through the “I-know-it-when-I-see-it” approach in identifying fatigue as a cause^1^; thus, establishing a national monitoring system will help improve uniformity in data collection and analysis between provinces, especially in rural areas. Although traffic flow characteristics have been found to have significant effects on the occurrence of crashes in many previous researches (Kerner 2013, 2015, 2016), they have not been considered here due to the incomplete or missing messages in TADS, so further works should pay close attention to the association between traffic flow conditions and probability of truck crash occurrence. All these suggestions and future studies might be helpful for promulgating potential policy initiatives and promoting effective strategies to increase overall safety performance on roads in China.

## Footnotes

1 The police officers generally ask the driver how long he or she’ been driving before the crash to determine whether the driver was fatigued at the time of the crash. Since each commercial long distance truck is officially required to be equipped with a dashboard camera in China, so the policy officers are able to examine the record of truck driver’s continuous driving to determine the influence of fatigue in crash. Additionally, the policy officers even can use traffic camera surveillances along the route to measure the point-to-point journey time according to the license plate so as to identify whether the truck driver is fatigued.
